# A case of a horseshoe appendix

**DOI:** 10.1186/s40792-016-0261-3

**Published:** 2016-11-23

**Authors:** Kazuya Takabatake, Jun Ikeda, Hirotaka Furuke, Chikage Kato, Takuya Kishimoto, Tatsuya Kumano, Kenichiro Imura, Katsumi Shimomura, Takeshi Kubota, Fumihiro Taniguchi, Yasuhiro Shioaki

**Affiliations:** Department of Surgery, Japanese Red Cross Kyoto Daiichi Hospital, 15-749 Honmachi, Higashiyama, Kyoto, Kyoto Japan

**Keywords:** Anomalies of the appendix, Horseshoe appendix

## Abstract

Anomalies of the appendix are extremely rare, and a horseshoe appendix is even rarer. A literature search has revealed only five reported cases. In this report, we present a case of a horseshoe appendix.

A 78-year-old man was referred for further examination following a positive fecal occult blood test. A mass in his ascending colon was detected on colonoscopy, while computed tomography showed that it was connected to the appendix. Tumor invasion derived from the ascending colon or appendix was suspected. We diagnosed ascending colon cancer prior to laparoscopic ileocecal resection. Macroscopic findings showed that the appendix connected to the back side of the mass, while microscopic findings showed that the mucosa and submucosa were continuous from the appendiceal orifice in the cecum to the other orifice in the ascending colon, where a type 1 tumor was observed on the orifice. We eventually diagnosed the patient with tubulovillous adenoma and a horseshoe appendix.

A horseshoe appendix communicates with the colon at both ends and is supplied by a single fan-shaped mesentery. Cases are classified by the disposal of the mesentery and the location of the orifice. Anatomical anomalies should be considered despite the rarity of horseshoe appendices.

## Background

Anomalies of the appendix are extremely rare. There have been several reports on the absence or duplication of the appendix. However, a literature search revealed only five reported cases of a horseshoe-shaped appendix [1–5]. In this report, we present a case of a horseshoe appendix that was incidentally found during resection of an adenoma in the ascending colon.

## Case presentation

**Fig. 1:**
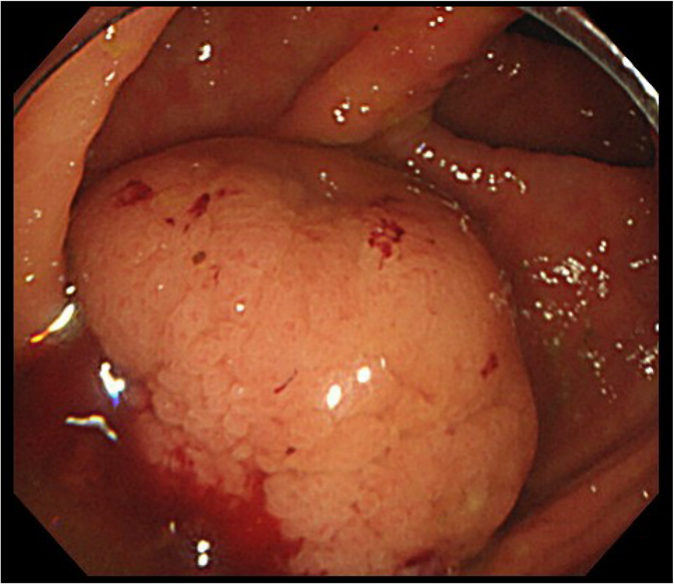
A type 1 mass detected in the ascending colon. Sub-mucosal invasion suspected from poor mobility

**Fig. 2:**
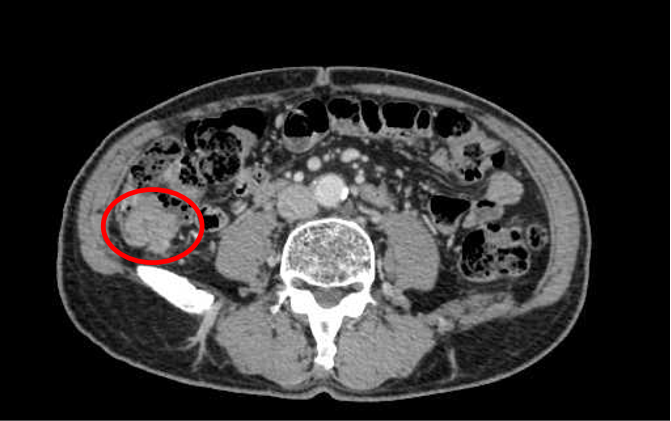
A 30-mm-wide mass in the ascending colon

**Fig. 3:**
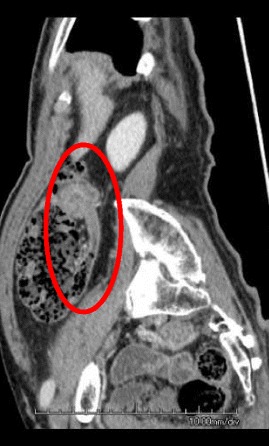
A mass connected with the appendix. Tumor invasion derived from the ascending colon or appendix suspected

**Fig. 4:**
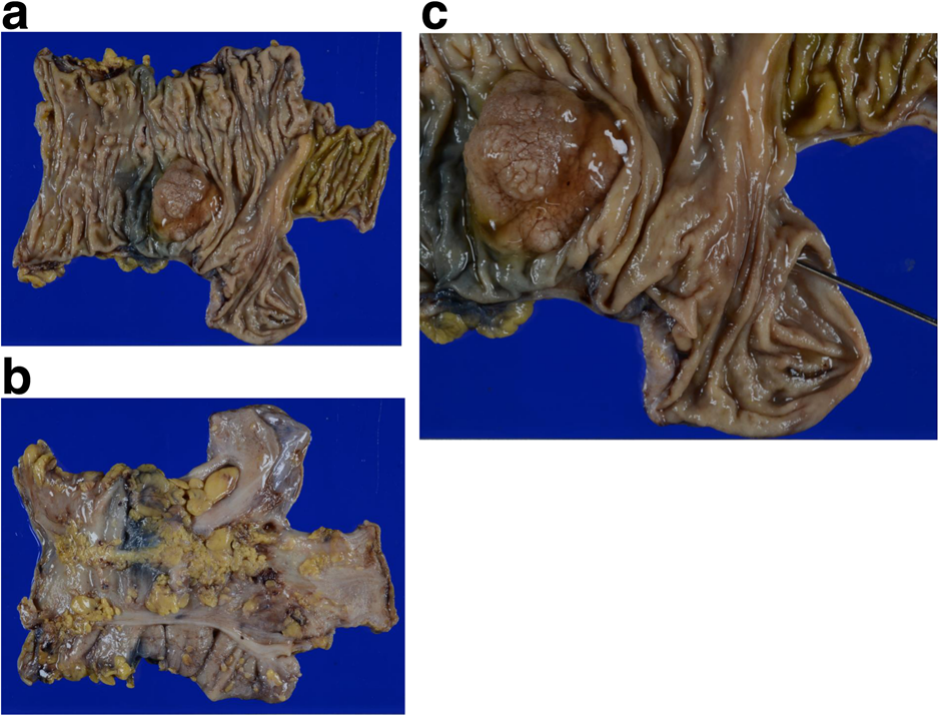
Resected specimen (**a**, **b)**. Appendix connected to the back side of the mass, inserting along the appendiceal orifice and reaching the adenoma of the ascending colon (**c)**

**Fig. 5:**
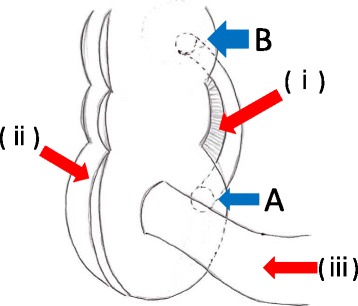
The diagram of the specimen. **a** The orifice of the cecum. **b** The orifice of the ascending colon. (*i*) The mesentery of the appendix, (*ii*) tenia of colon, (*iii*) the ileum

**Fig. 6:**
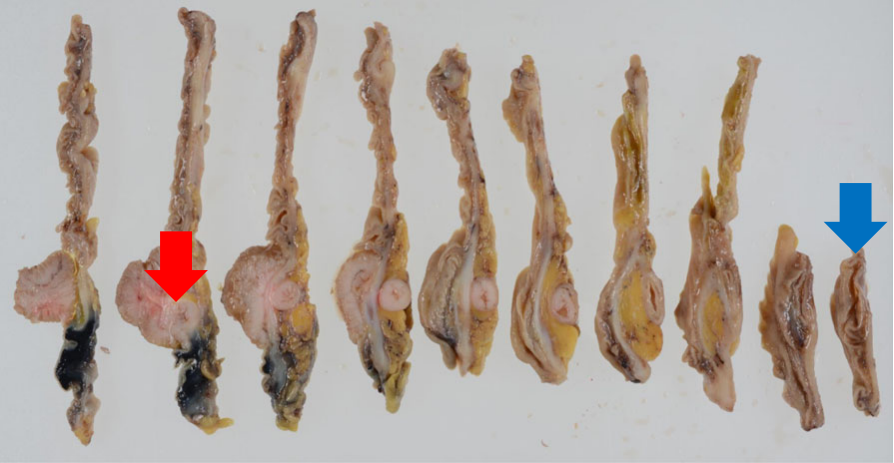
Mucosa and submucosa were continuous from the appendiceal orifice in the cecum to the other orifice in the ascending colon with a seamless muscular layer. A *blue arrow* is the orifice of the cecum, and a *red arrow* is the other of the ascending colon

A 78-year-old man was referred to us for further examination following a positive fecal occult blood test result. A mass that was possibly malignant was detected by colonoscopy in the ascending colon. There were no particular findings from physical examinations or hematological examinations, including the following tumor markers: cancer embryonic antigen and cancer antigen 19–9. Colonoscopy showed a type 1 mass in the ascending colon (Fig. 1) with submucosal invasion suspected from poor mobility. Computed tomography showed a 30-mm-wide mass in the ascending colon (Fig. 2) that was connected to the appendix. Tumor invasion derived from the ascending colon or appendix was suspected (Fig. 3). We preoperatively diagnosed ascending colon cancer, as follows: cT1, cN0, cM0, cStage1 (UICC/AJCC 7th). A standard laparoscopic ileocecal resection was then performed. Intraoperative findings showed that the appendix was connected to the ascending colon. It was suspected to be a tumor invasion and was therefore mobilized and resected carefully. Macroscopic findings showed the appendix connected to the back side of the mass, inserting along the appendiceal orifice and reaching the adenoma of the ascending colon (Figs. 4 and 5). Microscopic findings revealed that the mucosa and submucosa were continuous from the appendiceal orifice in the cecum to the other orifice in the ascending colon with a seamless muscular layer (Fig. 6). There was no evidence of inflammation or malignancy, and pathologically, the appendix was normal. There was a type 1 tumor on the orifice in the ascending colon, which was pathologically diagnosed as a tubulovillous adenoma with moderate atypia, along with an appendiceal extension. There was no evidence of lymph node metastasis. We finally diagnosed the patient with a tubulovillous adenoma and a horseshoe appendix. After undergoing the previously described surgery, the patient experienced a paralytic ileus and required fasting. He was discharged home on the 15th day after surgery.

### Discussion

Anomalies of the appendix are extremely rare. In a study by Collins, from among 50,000 appendix specimens, there were four cases of agenesis and two of duplication [6]. Duplications of the appendix were classified by Cave in 1936 [7] and modified by Wallbridge in 1963 [8] and Biermann in 1993 [9]. However, there were some cases that could not be classified using this classification (e.g., triplets of the appendix, horseshoe appendix).

Based on our review of the literature, our patient is the 6th reported case of a horseshoe-shaped appendix. Such an appendix is said to communicate with the colon at both ends and to be supplied by a single fan-shaped mesentery. We analyzed the five reported cases (Table. 1; our case plus the five previously reported), including four men and two women who ranged in age from 4 to 78 years (average 45). No case was diagnosed with a horseshoe appendix preoperatively, and the appendix was removed in all cases, including an ileocecal resection. The patients had no other anomalies and could be classified into two types based on the disposal of the mesentery and the location of the orifice: three frontal types, with the bases of the appendix located not on the tenia, and three sagittal types, with the bases along the tenia. The five previously reported cases showed that the appendix communicated with the cecum at both ends; only our case showed communication from the cecum to the ascending colon. There was no case in which an adenoma existed on the other orifice. There was one report in which a mucinous cystadenocarcinoma of the appendix invaded the ascending colon with fistula formation [10]. It could be argued that our case did not represent an anomaly of the appendix, but rather a fistula caused by an appendiceal neoplasm. However, we believe that our case represented a horseshoe appendix because the neoplasm on the other orifice was an adenoma, not a malignancy, and the mucosa and submucosa of the appendix were continuous, with a seamless muscular layer.

**Table 1:** Cases of a horseshoe appendix

Author	Year	Age	Sex	Diagnosis	Operation	Type	Orifice	Other anomalies	Detection of a horseshoe appendix
Mesko TW et al.	1989	33	Male	Sigmoid diverticulitis	Sigmoidectomy + appendectomy	Frontal	Cecum-cecum	None	Incidentally
DasGupta R et al.	1999	48	Male	Cecum perforation	Suturing perforation + appendectomy	Frontal	Cecum-cecum	None	Incidentally
Calotă F et al.	2010	43	Female	Appendicitis	Appendectomy	Sagittal	Cecum-cecum	None	Incidentally
Cem ORUÇ et al.	2013	64	Female	Appendicitis	Appendectomy	Sagittal	Cecum-cecum	None	Incidentally
Ch Gyan Singh	2016	4	Male	Appendicitis	Appendectomy	Sagittal	Cecum-cecum	None	Incidentally
Our case		78	Male	Adenoma in ascending colon	Laparoscopic ileocecal resection	Frontal	Cecum-ascending colon	None	Incidentally

Calota et al. reported a more complete classification system of the anomalies of the appendix [3], which we modified (Table 2).

**Table 2:** The classification of appendiceal anomalies

•Number anomalies
1. Agenesis: absence of appendix
2. Duplex appendix
A: partial duplication with both appendices sharing a common base like “Y-shaped” on a single cecum B: complete duplication of the appendix on a single cecum •B1 avian type: two appendices symmetrically placed on either side of the ileocecal valve •B2 tenia-coli cecum type: one appendix arising from the usual site of the cecum and the other arising from the cecum along the tenia •B3 tenia-coli hepatic flexure type: one appendix arising from the usual site of the cecum and the other arising from the hepatic flexure of the colon along the tenia. •B4 tenia-coli splenic flexure type: one appendix arising from the usual site of the cecum and the other arising from the splenic flexure of the colon along the tenia. C: duplication of the cecum, each having its own appendix
3. Triplex appendix: complete triplication of appendix on the cecum
• Shape anomalies Horseshoe Appendix Disposal of the mesentery • Sagittal disposal: the both bases of the appendix along the tenia in sagittal direction • Frontal disposal: the bases of the appendix not on the tenia Location of the orifice • Cecum-cecum • Cecum-ascending colon

In this classification, anomalies of the appendix are classified by number (e.g., agenesis, duplication, and triplet) and shape (e.g., horseshoe), while anomalies of the horseshoe appendix are further classified by the disposal of the mesentery and the location of the orifice.

## Conclusions

Although most surgeons will not experience anomalies of the appendix, including the horseshoe appendix, anatomical anomalies of appendix should nevertheless be considered, despite their rarity.
